# Relevance of retrovirus quantification in cerebrospinal fluid for neurologic diagnosis

**DOI:** 10.1186/s12929-015-0170-y

**Published:** 2015-08-08

**Authors:** Carolina Rosadas, Marzia Puccioni-Sohler

**Affiliations:** Cerebrospinal Fluid Laboratory, Hospital Universitário Clementino Fraga Filho, Universidade Federal do Rio de Janeiro, Rua Professor Rodolpho Paulo Rocco 255, 3°andar, Rio de Janeiro, 21941-913 Brazil; Neuroinfection Unit, Hospital Universitário Gaffrée e Guinle, Universidade Federal do Estado do Rio de Janeiro, Rua Mariz e Barros, 775, Rio de Janeiro, 20270-004 Brazil; Laboratório de Líquido Cefalorraquiano, Serviço de Patologia Clínica, Hospital Universitário Clementino Fraga Filho, Universidade Federal do Rio de Janeiro, Rua Professor Rodolpho Paulo Rocco 255, 3 ± andar, Rio de Janeiro, RJ 21941-913 Brazil

**Keywords:** Retrovirus, Cerebrospinal fluid, Viral load, Proviral load, HTLV, HIV

## Abstract

Different human retroviruses, such as Human Immunodeficiency Virus (HIV) and Human T-cell Lymphotropic Virus (HTLV), can cause neurologic infection. However, a definitive diagnosis may be hampered by several factors. Quantification of the viral or proviral load in cerebrospinal fluid (CSF) may be helpful in the diagnosis of nervous system disorders due to retroviral infection and may influence the treatment approach. The present work discusses retrovirus infection and neurologic impairment, as well as the usefulness of the determination of the HIV and HTLV proviral or viral load in cerebrospinal fluid in cases of neurologic disorder, in light of recent advances in this field. This study also discusses the different molecular techniques for quantifying the proviral load (real-time quantitative PCR, droplet digital PCR, and semi-nested real-time reverse transcription PCR) that are currently available.

## Introduction

The *Retroviridae* family comprises enveloped RNA viruses that can infect several hosts. The viral particle presents the enzymes reverse transcriptase and integrase. Thus, after the reverse transcription, the proviral DNA can be integrated into host cell genome [1]. In this context, molecular assays can be used to determine the presence and quantity of both viral RNA and proviral DNA in the infected host cell [2–5]. Human retroviruses, such as Human Immunodeficiency Virus (HIV) and Human T-cell Lymphotropic Virus (HTLV), can cause neurologic infection [6–12]. However, in these cases, a definitive diagnosis can be very challenging and helpful for the early treatment. The difficulty is associated with distinct factors, such as the wide range of symptoms that can be observed in both types of infection and the similarity of those symptoms with those of other diseases. This problem occurs not only in developing countries, where there is a high prevalence of infectious agents, but worldwide. The presence of coinfections and the possibility of secondary nervous system damage due to dual infection are other factors that can complicate the diagnosis [11, 13–16]. The presence of virus in the central nervous system (CNS) of individuals without neurologic alterations also hampers a conclusive diagnosis. Therefore, in cases of CNS impairment, such as HTLV-1–associated myelopathy and HIV-associated dementia, viral quantification in cerebrospinal fluid (CSF) may clarify the diagnosis [8, 13, 17–22].

In the current study, we discuss the usefulness of HIV and HTLV-1 quantification in CSF among patients with neurologic disorders due to retrovirus infection, on the basis of the recent knowledge achieved in this field.

## Review

### Retrovirus infections and neurologic impairment

HTLV-1 can successfully infect the central nervous system (CNS). Although the great majority of HTLV-1 infected individuals remain asymptomatic throughout their lifetime, about 5 % may present a chronic, incapacitating neurologic disorder [10, 23, 24]. This disorder, called HTLV-1–associated myelopathy/tropical spastic paraparesis (HAM/TSP), is characterized predominantly by spinal cord damage associated with HTLV-1 infection [10, 25].

Regarding HIV infection, severe neurocognitive conditions, usually resulting in death, have been detected in HIV-infected individuals since the beginning of the HIV epidemic [6, 26]. This alteration was observed in persons with advanced HIV-1 infection and was called HIV-Associated Dementia (HAD) [27]. A few years later, milder forms of neurocognitive impairment in HIV-1-infected persons were reported, occurring even before the onset of advanced systemic disease [28]. After the introduction of combination antiretroviral therapy (cART), the incidence of HAD decreased dramatically [29]. However, milder forms of HIV-associated neurologic disorders became highly prevalent. Thus, a new denomination was suggested: HIV-associated neurocognitive disorders (HAND) [7, 27]. According to this new terminology, HAND can be further classified into asymptomatic neurocognitive impairment (ANI), mild neurocognitive disorder (MND), and HIV-associated dementia (HAD). HAND is estimated to affect up to 50 % of HIV-infected individuals [7, 27]. It is important to note that the prevalence of HAD decreased in the cART era, but for mild to moderate HAND forms. In fact, the prevalence of HAND in HIV-infected patients without AIDS increased from 29 to 36 % after the implementation of combined antiretroviral therapy. There was no significant difference in the prevalence of HAND among AIDS patients [9].

### HTLV proviral load in CSF

According to WHO guidelines for HAM/TSP diagnosis, a patient can be classified under definitive HAM/TSP in the presence of chronic progressive spastic paraparesis associated with antibodies detection in both blood and CSF. Some individuals, however, do not present antibodies in CSF or do not present the classical symptoms of disease, despite the presence of antibodies in both compartments. Such individuals are classified under probable HAM/TSP [30]. Moreover, in cases of recent infection and of passive transfer of antibodies (as can occur in vertical transmission), antibodies detection is not recommended due to false-positive or false-negative results [31]. It is important to highlight that antibodies can be passively transferred through the blood-CSF barrier even in asymptomatic individuals [17].

The detection of HTLV-1 antibodies is done by screening (ELISA) and confirmatory tests (Western blot). Some patients can present indeterminate or discordant Western blot results [32, 33]. The same can occur in HIV diagnosis [34]. In this context, tests that are able to detect and quantify viral genome can be very useful. The HTLV-1 proviral load (PVL) in blood is higher in HAM/TSP patients than in asymptomatic carriers. However, previous studies have failed to determine a reliable cutoff value for an accurate HAM/TSP diagnosis [35, 36].

Regarding the PVL in CSF, patients with HAM/TSP were found to present a higher PVL compared with asymptomatic HTLV-1–infected individuals [13, 17, 20, 21, 37]. Moreover, in HAM/TSP patients, the PVL CSF/PVL blood ratio was always higher than 1 in HAM/TSP and lower than 1 in AC [38] PVL in CSF inverse correlated with intrathecal synthesis of HTLV-1 antibodies (HTLV-1 AI) [12]. Another interesting point is that PVL in CSF combined to intrathecal synthesis of HTLV-1 antibodies analysis showed to be useful in discriminating between HAM/TSP and multiple sclerosis (MS) [13]. This is extremely important because HTLV and MS present very similar symptoms.

Therefore, the quantification of the PVL in CSF may be a good marker for HAM/TSP diagnosis, mainly when associated with other tests, such as HTLV-1 AI.

### HIV viral and proviral load in CSF

The determination of the HIV viral load in blood is widely used to assess the disease progression and the response to antiretroviral therapy. As previously mentioned, HIV can infect the CNS, and HAND can be observed even in patients with adequate control of viral replication in plasma [7–9, 18, 22, 39]. The mechanism that leads to the persistence of neurologic impairment in patients with suppressed viral replication in the bloodstream is poorly understood. Some authors consider chronic inflammation, the presence of coinfections (such as HCV), drug abuse, aging, and antiretroviral drug effects as factors that can contribute to the persistence of HAND [15]. In this context, HIV compartmentalization might play an important role in nervous system damage. To understand HIV compartmentalization and its association with HAND development, as well as acquired drug resistance, some basic concepts of viral biology are discussed.

**Fig. 1:**
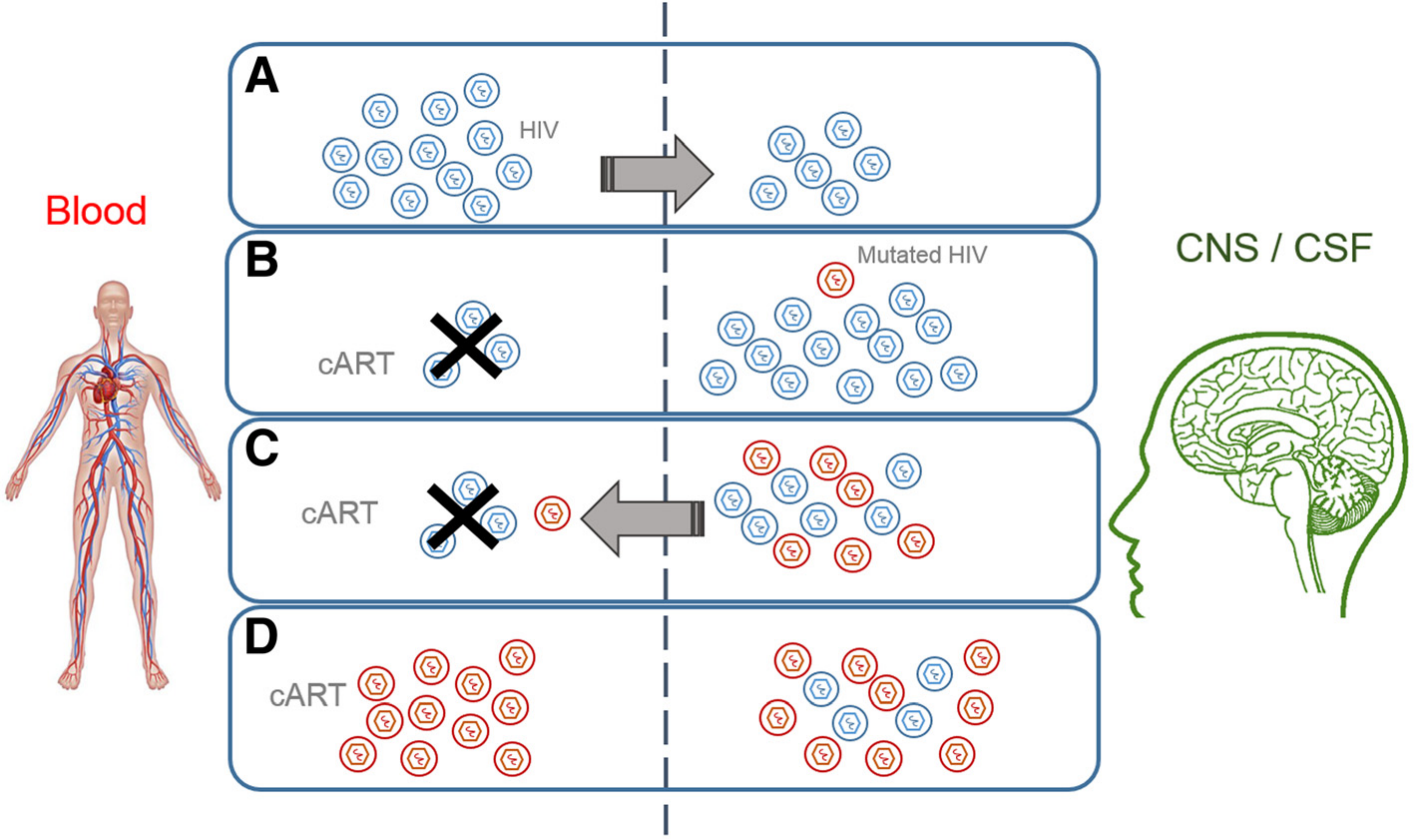
Mechanism of HIV-acquired drug resistance with the central nervous system as reservoir. **a** Initial phase of HIV infection: HIV is replicating in blood and access the CNS. **b** cART is initiated and controls the viremia. However, depending on the drug regimen, the drug access to the CNS is poor, and HIV can efficiently replicate in this reservoir. As replication occurs, a mutated virus can be formed (compartmentalization). **c** Mutated HIV enters the bloodstream. The mutated virus is able to replicate more efficiently in the cART poor environment (CNS) and could get into the bloodstream. **d** The mutant drug-resistant virus replicates efficiently even with cART therapy. In such cases, the medication has to be changed. The higher replication rate in the CNS is associated with the high proviral load in the CNS despite the proviral load in blood and is associated with neurologic abnormalities in HIV-infected patients

HIV presents a high mutation rate, which can result in the coexistence of viral quasispecies within a host. These quasispecies evolve as a result of selection pressures, such as those imposed by antiviral therapy, and can be restricted to cells or tissues (compartmentalization) [40]. Previous studies have described the HIV compartmentalization in the CNS, which leads to selective replication of distinct HIV quasispecies, resulting in resistance confined to the CNS [19, 41]. It has also been pointed out that the mutated virus may cause further viremia and may be related to resistance to cART; Fig. 1 presents this mechanism. A recent study showed that HIV-infected individuals presented more resistant virus in CSF than in blood. This occurred in both antiretroviral-naïve and treated patients, being more frequent in the latter [42]. The administration of drugs that are poorly distributed in the CNS is a factor that can contribute to the development of drug-resistant virus in the nervous system. Suboptimal adherence to the treatment regimen may also lead to insufficient drug concentration in CSF, which may also contribute to viral resistance [3, 8].

Thus, several studies have shown that HIV can escape in CSF despite viral suppression in blood [3, 8, 18, 22, 43]. This evasion can contribute to discordant viral loads and to a high viral load in CSF. A study of the HIV proviral load in blood and CSF among HIV-seropositive individuals with and without HAND found that the CSF proviral load was greater in individuals with HAND than in those without HAND. Moreover, the PVL in seropositive patients was greater in CSF than in blood. The proviral load was also observed to be greater in the blood and CSF of subjects with more advanced systemic disease and HAND [5]. Other studies have corroborated these findings, showing a high viral load in CSF among patients with HAD [44].

These data highlight the importance of the quantification of the proviral or viral load in CSF among HIV-infected patients presenting neurologic disturbance and/or drug resistance. In such cases, genotyping tests of CSF virus are extremely important. In fact, some studies have reported successful genotyping in CSF in cases in which it was not possible to apply genotyping in blood [45].

### Antiretroviral treatment and the proviral load

Previous studies have reported the effect of antiretroviral treatment on the HIV proviral load. In particular, the proviral load in CSF was shown to decrease after the implementation of a therapeutic regimen with drugs with improved CNS penetration [43].

Indeed, the drug concentration in the CNS seems to be crucial for viral control in the nervous system. A recent study tested two different therapeutic regimens with distinct concentrations of darunavir. Only two patients presented detectable viral genome in CSF, and those two had the lower concentration of the drug in CSF despite the prescribed dosage of orally administered drug, confirming that CSF assessment is an important issue [46].

Some drugs used as monotherapy, such as lopinavir/ritonavir and zidovudine, had a significant effect on decreasing CSF replication. In contrast, didanosine and saquinavir were not able to control viral replication in CSF. Abacavir was also tested as an adjunctive therapy in patients presenting HAD, with no effect in CSF HIV RNA. In this context, a CNS penetration-effectiveness (CPE) score was proposed. According to the CPE, the antiretrovirals are scored from 1 to 4 (with 4 indicating the most neuro-effective drug). This is determined based on the drug characteristics, pharmacokinetic features, and pharmacodynamic properties. The composite CPE can be calculated by summing single drug scores to obtain the treatment score. Usually, a higher CPE score is associated with a lower viral load in CSF [47, 48].

Another important point associated with the successful control of viral replication in CSF is related to viral resistance. Thus, the therapeutic regimen should be modified according to the results obtained by genotypic resistance testing in CSF samples.

Regarding HTLV infection, the specific therapy for HAM/TSP remains very disappointing, and symptomatic treatment is still the most commonly used therapy [10]. There are few studies showing the impact of therapy on the CSF PVL of HAM/TSP individuals. In one report, after the corticosteroid therapy, the PVL in blood decreased, but the PVL in CSF remained unchanged. The patient presented an acute onset, a rapid progression, and repeated exacerbation of neurologic symptoms, which is not the classical presentation of HAM/TSP [20].

### Molecular assays to determine the proviral and viral load

Currently, quantitative real-time polymerase chain reaction (qPCR) and reverse transcription (RT)-qPCR are the standard and most commonly used techniques to determine the proviral or viral load [37, 49, 50]. qPCR is known to have high sensitivity, which is associated with the ability to amplify the target gene, and high specificity, which is related to primer annealing. In this context, there are two main systems for the identification of the sign: the use of fluorometric probes (such as the TaqMan system) and the use of intercalating dyes (such as SYBR Green). The use of a fluorometric probe increases the assay specificity once the probe hybridizes to a complementary target sequence in the target DNA, but not to nonspecific PCR products or primer dimers, which may happen when intercalating dyes are used [51, 52]. However, qPCR presents limitations that can influence the accuracy of the test. The dependence on a standard curve, for example, may result in high inter-assay variability, mainly in compartments with a low number of cells, such as CSF [53].

In this context, a novel technique, droplet digital PCR (ddPCR), can also be used for viral or proviral load quantification. The advantage of this technique is that it does not require a standard curve. In fact, ddPCR allows the direct absolute quantification of a target gene, which can positively influence the accuracy of the PVL quantification. In a recent study, ddPCR presented low intra- and inter-assay variability in the determination of the HTLV-1 PVL in CSF of infected patients. The same study concluded that, regarding the HTLV-1 proviral load in peripheral blood mononuclear cells (PBMCs), the inter-assay variability of ddPCR was lower compared with qPCR [37]. It is important to note, however, that ddPCR presented false-positive signals when it was used to detect the viral load in HIV-infected individuals [54].

Regarding HIV infection, because some individuals can present viral replication suppressed below the detection limit of the available diagnostic methods [39], some authors have suggested the use of a semi-nested real-time reverse transcription PCR assay to determine the plasma viral load. In this technique, two successive PCR reactions are done to increase the sensitivity of the test and, consequently, reduce the limit of detection [55]. Thus, semi-nested RT-qPCR may be an interesting alternative in patients with a low viral load. Nevertheless, no study has applied this technique in CSF.

Regardless of the chosen technique, prior validation is essential, that is, before implementation in CSF routine analysis. Cross-validation by multiple laboratories is indicated.

## Conclusions

The determination of the viral or proviral load in CSF of HTLV-1– and HIV-infected individuals with neurologic disease may be a good marker for diagnosis, considering the proximity of the lesion and the local infection. The association of PVL measurement with other tests, such as PVL in blood and intrathecal synthesis of specific antibodies analysis in HTLV infection and HIV genotyping, might bring an important contribution to clinicians. In some cases, it can make the difference for a conclusive diagnosis or even to therapeutic approach. However, prior validation of molecular assays before implementation in laboratorial routine is essential, mainly regarding the CSF examination. Many laboratories use the same methodology applied in blood, forgetting that CSF is a body fluid with different constituents that may interfere in laboratory tests. Even greater emphasis should be given to HTLV, which, being a neglected disease, does not have approved commercial kits for use in CSF testing; thus, “in-house” tests are mandatorily used. In this case, the international standardization of tests would be beneficial for the acquisition of reliable and comparable results that would make possible a better clinical interpretation of the laboratory findings of patients.
